# CT perfusion based ASPECTS improves the diagnostic performance of early ischemic changes in large vessel occlusion

**DOI:** 10.1186/s12880-021-00593-5

**Published:** 2021-04-12

**Authors:** Tiegong Wang, Luguang Chen, Xianglan Jin, Yuan Yuan, Qianwen Zhang, Chengwei Shao, Jianping Lu

**Affiliations:** 1 Department of Radiology, Changhai Hospital of Shanghai, Navy Medical University (Second Military Medical University), No. 168 Changhai Road, Shanghai, 200433 China; 2 Department of Cardiac Surgery, Shanghai Tenth People’s Hospital, Tongji University School of Medicine, No. 301 Yanchang Middle Road, Shanghai, 200072 China

**Keywords:** Stroke, Perfusion imaging, Tomography, X-ray computed, Prognosis

## Abstract

**Background:**

ASPECTS scoring method varies, but which one is most suitable for predicting the prognosis still unclear. We aimed to evaluate the diagnostic performance of Automated (Auto)-, noncontrast CT (NCCT)- and CT perfusion (CTP) -ASPECTS for early ischemic changes (EICs) in acute ischemic stroke patients with large vessel occlusion (LVO) and to explore which scoring method is most suitable for predicting the clinical outcome.

**Methods:**

Eighty-one patients with anterior circulation LVO were retrospectively enrolled and grouped as having a good (0–2) or poor (3–6) clinical outcome using a 90-day modified Rankin Scale score. Clinical characteristics and perfusion parameters were compared between the patients with good and poor outcomes. Differences in scores obtained with the three scoring methods were assessed. Diagnosis performance and receiver operating characteristic (ROC) curves were used to evaluate the value of the three ordinal or dichotomized ASPECTS methods for predicting the clinical outcome.

**Results:**

Sixty-three patients were finally included, with 36 (57.1%) patients having good clinical outcome. Significant differences were observed in the ordinal or dichotomized Auto-, NCCT- and CTP-ASPECTS between the patients with good and poor clinical outcomes (all *p* < 0.01). The areas under the curves (AUCs) of the ordinal and dichotomized CTP-ASPECTS were higher than that of the other two methods (all *p* < 0.01), but the AUCs of the Auto-ASPECTS was similar to that of the NCCT-ASPECTS (*p* > 0.05).

**Conclusions:**

The CTP-ASPECTS is superior to the Auto- and NCCT-ASPECTS in detecting EICs in LVO. CTP-ASPECTS with a cutoff value of 6 is a good predictor of the clinical outcome at 90-day follow-up.

## Background

Cerebrovascular disease is the second leading cause of death worldwide, and ischemic stroke accounts for approximately 50% of the 6.5 million deaths each year, and stroke has become the main reason of mortality in China [1, 2]. The Alberta Stroke Program Early CT Score (ASPECTS) is a noncontrast CT (NCCT)-based scoring system that can quantitatively evaluate the early ischemic changes (EICs) of acute ischemic stroke (AIS) in the blood supply territory of the middle cerebral artery (MCA) [3]. It is a simple and feasible method that can be performed in both primary and comprehensive stroke centers [3]. However, limitations of the ASPECTS in evaluating EICs are its low consistency and repeatability among observers [4]. In the early stage of ischemic stroke, the density changes on NCCT images are so weak that the reader cannot easily identify them. Moreover, there are differences in evaluating the ischemia territory between different observers, resulting in poor reliability and consistency for NCCT-ASPECTS.

Recently, with the rapid development of artificial intelligence and deep learning technologies, automatic ASPECTS scoring has been used to minimize intra- and interobserver variability and improve its accuracy [5, 6]. At present, a few commercial software programs can be used to obtain automated ASPECTS (Auto-ASPECTS), which showed good agreement with readers [7–9]. However, there is still controversy about the utility of the ASPECTS in selecting patients for endovascular treatment [10]. One study showed that in AIS patients with anterior circulation large vessel occlusion (LVO), the degree of EICs on NCCT before treatment can predict the clinical outcome and risk of intracranial hemorrhage (ICH) after reperfusion [10]. Compared with NCCT, CT perfusion imaging (CTP) can provide both hemodynamic and physiological information of ischemic brain tissue and even show EICs better which will benefit for the determining of treatment strategies. Recently, several studies reported that qualitative assessment of the ASPECTS using CTP imaging (CTP-ASPECTS) showed a better potential in improving the accuracy of detecting EICs, reducing the variability among observers, and predicting the outcome of patients' clinical function [11–13]. However, comparisons of the Auto-, NCCT- and CTP-ASPECTS in predicting the clinical outcome of patients with AIS have not been explored thus far.

Therefore, the purpose of this study was to compare the diagnostic performance of the Auto-, NCCT- and CTP-ASPECTS for EICs in AIS patients with LVO, and to explore which scoring method is most suitable for predicting the clinical outcome.

## Methods

### Patients

This retrospective study was approved by the Ethics Committee of Changhai Hospital of Shanghai (IRB protocol number: CHEC2013-204) and written informed consent was waived. Between January 2019 and June 2019, eighty-one consecutive patients (44 males and 37 females; median age, 72 years; range 64–80 years) with AIS and intracranial LVO were enrolled in this study. The inclusion criteria were as follows: (1) time interval between symptom onset and admission less than 24 h; (2) complete multimodal CT scan within 20 min after admission, including NCCT, CT angiography (CTA), and CTP; and (3) occlusion of the intracranial internal carotid artery (IICA) or MCA (M1 and M2). Patients were excluded using the following criteria: (1) undiagnosable NCCT images due to motion artifacts; (2) occlusion of the anterior cerebral or posterior circulatory arteries; and (c) incomplete clinical data collected at 90-day follow-up. Demographic characteristics were recorded, including patient age, gender, baseline National Institute of Health Stroke Scale (NIHSS) score, onset-to-door time, hyperdense MCA sign, location of LVO, number of patients with different treatment strategies (antiplatelet therapy, mechanical thrombectomy and intravenous thrombolysis), and collateral circulation. For patients who received mechanical thrombectomy, successful recanalization was defined as modified Thrombolysis in Cerebral Infarction Score (mTICI) 2b or 3.

### CT protocol

All imaging was performed on a 256-slice CT scanner (Brilliance iCT Elite, Philips healthcare, the Netherlands). Parameters for helical NCCT was 120 kV, 350 mAs, thickness = 5 mm, slices = 30, field-of-view (FOV) = 250 × 250 mm^2^, and matrix = 496 × 496. The main imaging parameters of the CTP were 80 kV, 180 mAs, whole brain coverage in the z-axis, FOV = 220 × 220 mm^2^, matrix = 512 × 512, slice thickness = 5 mm, JOG scanning mode, and 14 consecutive phases acquired with a temporal resolution of 4 s. A total of 50-ml of iobitridol (Xenetix-350; Guerbet, France) was intravenously injected at a speed of 5-ml/s, followed by a 20-ml saline flush at 5 ml/s. Parameters for CTA were 120 kV, 300 mAs, FOV = 220 × 220 mm^2^, matrix = 512 × 512, thickness = 1 mm, and slices = 399. The same protocol for the injection of contrast agent of CTP was used here, except for the use of 45-ml of contrast.

### Image analysis

**Fig. 1:**
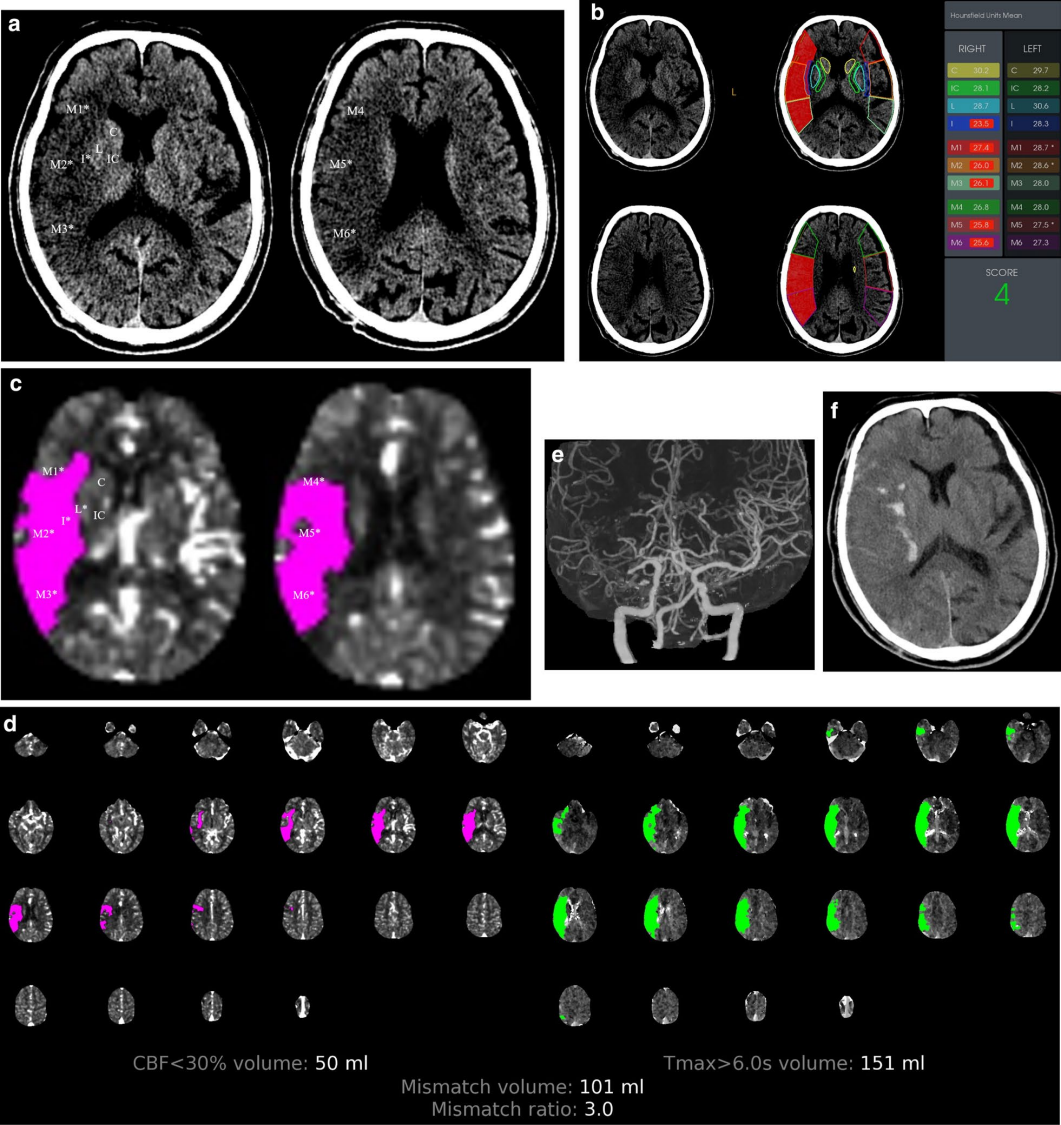
A 75-year-old male patient was sent to the emergency department 8 h after the onset of symptoms with a baseline NIHSS score of 22. The NCCT-, Auto- and CTP-ASPECTS were 4, 4, and 2 points (**a**–**c**), respectively. CT perfusion revealed an infarct core of 50 ml, hypoperfusion of 151 ml, mismatch volume of 101 ml, and mismatch ratio of 3.0 (**d**). The maximum intensity projection showed that the proximal M1 segment of the right middle cerebral artery was occluded (**e**). The patient successfully underwent mechanical thrombectomy 65 min after admission. A review of the NCCT 14 h after revascularization revealed a large infarction of the right temporal lobe with intracranial hemorrhage (**f**). The patient had a poor clinical outcome at the 90-day follow-up. * indicates the regions of the early ischemic changes of the 10 defined middle cerebral artery (MCA) vascular territories. C = caudate head, I = insula, IC = internal capsule, L = lentiform nucleus, M1 = frontal operculum, M2 = anterior temporal lobe, M3 = posterior temporal lobe, M4 = anterior MCA, M5 = lateral MCA, M6 = posterior MCA

**Fig. 2:**
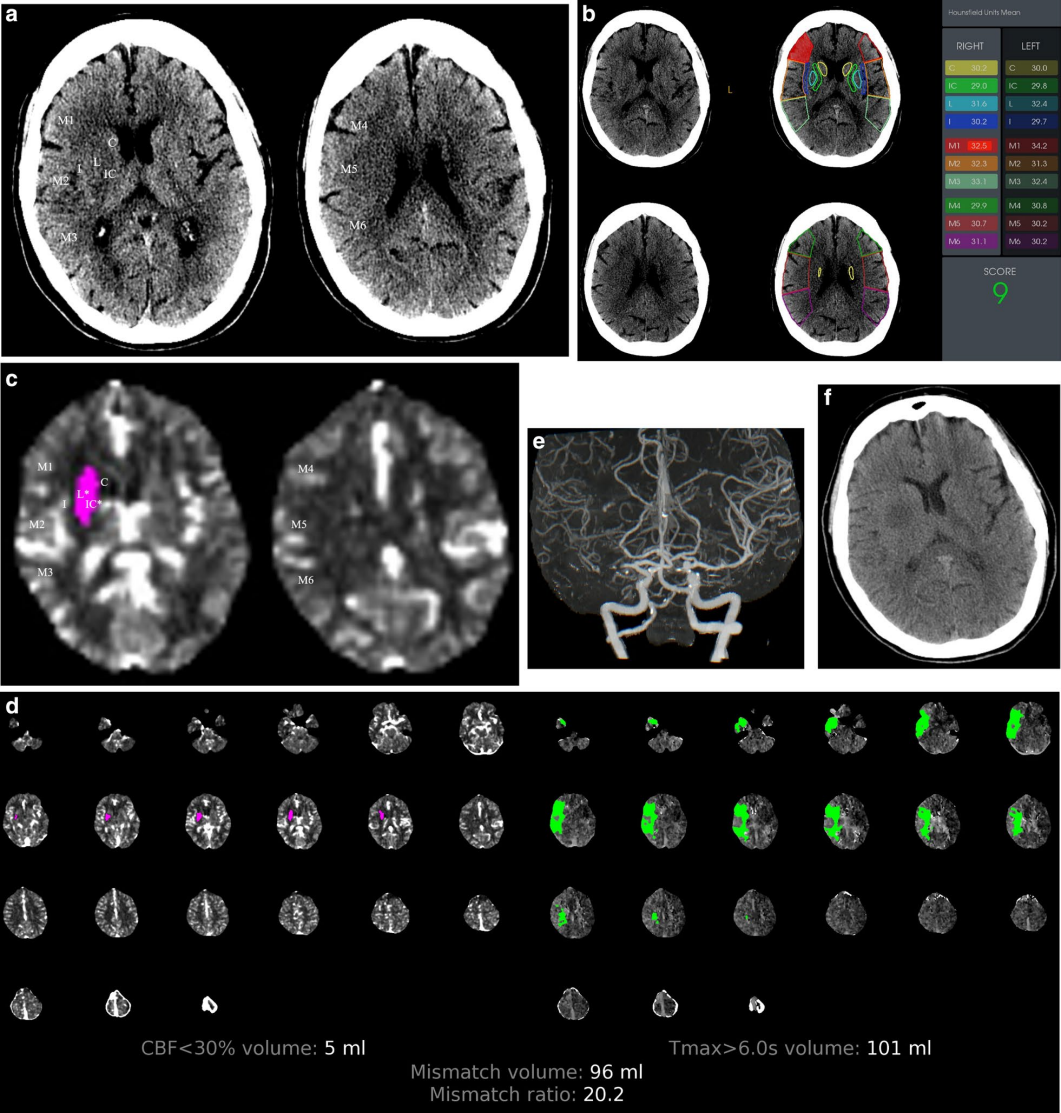
An 80-year-old female patient arrived at the hospital 1.5 h after the onset of symptoms, with a baseline NIHSS score of 20. The NCCT-, Auto- and CTP-ASPECTS were 10, 9, and 8 points (**a**–**c**), respectively. CT perfusion showed an infarct core of 5 ml, hypoperfusion of 101 ml, mismatch volume of 96 ml, and mismatch ratio of 20.2 (**d**). The Auto-ASPECT showed ischemia in the M1 region, while the CTP-ASPECT showed ischemia in the internal capsule and the lentiform nucleus region. The maximum intensity projection showed that the proximal M1 segment of the middle cerebral artery was occluded (**e**). The infarct core shown by CT perfusion was equivalent to the final infarct volume shown by NCCT reviewed 16 h after successful mechanical thrombectomy (**f**). The patient had a good clinical outcome at the 90-day follow-up. * indicates the regions of the early ischemic changes of the 10 defined middle cerebral artery (MCA) vascular territories. C = caudate head, I = insula, IC = internal capsule, L = lentiform nucleus, M1 = frontal operculum, M2 = anterior temporal lobe, M3 = posterior temporal lobe, M4 = anterior MCA, M5 = lateral MCA, M6 = posterior MCA

All reconstructed images were automatically sent to picture archiving and communication system (Version 3.0, GE Healthcare, Milwaukee, WI) and a RAPID server (Version 4.9; iSchemaView, Menlo Park, California) for diagnosis and further processing. Two radiologists (T.W. and Y.Y. with > 10-year of experience in neuroradiology) who were blinded to each patient’s clinical data read the NCCT images, with a window level of 35 and window width of 30, by consensus to calculate the NCCT-ASPECTS (Figs. 1a, 2a)[14]. Quantitative perfusion parameters were automatically quantified using RAPID, including the volumes of the infarct core, hypoperfusion and relative cerebral blood volume (rCBV) < 38%, time until residue function reached its peak (Tmax) > 10 s, mismatch volume (MMV), mismatch ratio (MMR), and hypoperfusion index (HI). The infarct core was defined as the region with relative cerebral blood flow (rCBF) < 30% of normal tissue, and hypoperfusion was defined as tissue with Tmax > 6 s. Collateral circulation was evaluated according to the Miteff collateral score [15]. MMV was calculated by subtracting the volume of rCBF < 30% from that of Tmax > 6 s, MMR was the ratio of the volumes of Tmax > 6 s and rCBF < 30%, and HI was the ratio of the volumes of Tmax > 10 s to Tmax > 6 s. Auto-ASPECTS was automatically determined on NCCT using RAPID, with the ischemic territory marked in red, and the scoring result displayed (Figs. 1b, 2b) [7]. CTP-ASPECT was evaluated by a third radiologist, with > 15 years of work experience in neuroradiology. The steps are as follows: firstly, the mismatch map was selected from the output images of RAPID, then using the left column (map of rCBF) of the mismatch map to perform the ASPECTS, of which the pink regions (rCBF < 30%, Fig. 1c, 2c) showed on the mismatch map, lastly, the score was assessed by those regions corresponding to the 10 ASPECTS areas on each hemisphere (Figs. 1c, 2c).

### Clinical outcome

The primary clinical outcome was assessed by the 90-day modified Rankin scale (mRS) score. The mRS score ranged from 0 (no symptom) to 6 (death). Good clinical outcome was defined as mRS score of 0–2 at 90-day. Secondary outcome included symptomatic ICH and stroke related death.

### Statistical analysis

Statistical analysis was carried out using SPSS software (Version 19, IBM Statistical Package for the Social Sciences, Chicago, IL). Quantitative data are presented as the means ± standard deviations or medians (interquartile ranges: IQRs) depending on the normality of the distribution of continuous variables, and categorical data are expressed as percentages. Clinical characteristics and perfusion parameters were compared between patients with good and poor clinical outcomes using the Mann–Whitney U test or chi-squared test when appropriate. Differences in scores made by the three scoring methods were assessed using related-samples Friedman’s two-way analysis of variance by ranks or Cochran’s Q test, and post hoc multiple comparisons were performed with Bonferroni correction. Sensitivity, specificity, negative and positive predictive values, and receiver operating characteristic (ROC) curves were used to evaluate the value of ordinal and dichotomized ASPECTS in the three scoring methods for predicting the clinical outcome. The cut off value was determined by the maximum Youden index (= sensitivity + specificity − 1) under the ROC curve. A *p* value of < 0.05 was considered statistically significant.

## Results

### Patients

Eighty-one AIS patients with intracranial LVO were enrolled. Among these patients, 18 were excluded for the following reasons: 2 patients had insufficient image quality for further assessment, 8 patients had occlusion of the anterior and posterior arteries, and 8 patients didn’t participate in the follow-up evaluation. Finally, 63 patients with a median age of 70 (IQR 66–78) were finally included. All patients were divided into the following groups: 36 (57.1%) and 27 (42.9%) patients with good and poor clinical outcomes, respectively. 13 patients received medical management only, including patients treated with antiplatelet therapy and intravenous thrombolysis. Totally, 50 patients received mechanical thrombectomy, the median time of imaging to groin puncture was 50 min, ranging from 7 to 185 min, among them 47 patients were successfully recanalized. 3 of 50 patients with failed recanalization had poor clinical outcome, while 30 of 50 patients with successful recanalization had good clinical outcome and the other 17 successful recanalization had poor clinical outcome.

### Clinical characteristics and perfusion parameters

Baseline clinical characteristics and CTP parameters of the patients with good and poor clinical outcomes are summarized in Table 1. The median (IQR) age of the patients with good and poor clinical outcomes was 68 (65, 77) and 71 (66, 81), respectively. Thirty-seven (58.7%) patients had an occlusion at MCA M1, and 34 (54.0%) had left-side stroke. Thirty-six (57.1%) patients had an mRS ≤ 2, and 7 (11.1%) patients were died at the 90-day follow-up. There were significant differences in the baseline NIHSS score, poor collateral circulation, infarct core volume, the volumes of rCBV < 38%, hypoperfusion and Tmax > 10 s, MMR and HI (all *p* values < 0.05) between the patients with good and poor clinical outcomes.

**Table 1:** Clinical characteristics and CT perfusion parameters in patients with good and poor clinical outcomes

Characteristics	Overall (*n* = 63)	Good clinical outcome (*n* = 36)	Poor clinical outcome (*n* = 27)	*p* value
Age^*^, year	70 (66, 78)	68 (65, 77)	71 (66, 81)	0.1406
Female^$^	29 (46.03)	18 (50.00)	11 (40.74)	0.4656
Onset-to-door time^*^, *h*	3.0 (2.0, 5.0)	2.5 (2.0, 6.0)	2.0 (1.5, 5.0)	0.1966
Baseline NIHSS score^*^	19 (11, 22)	13.50 (5, 20)	20 (19, 30)	0.0005
Hyperdense MCA sign^$^	17 (26.98)	8 (22.22)	9 (33.33)	0.3255
Left site stroke^$^	34 (53.97)	19 (52.78)	15 (55.56)	0.8267
Occlusion site^$^				
IICA	14 (22.22)	6 (16.67)	8 (29.63)	0.2608
MCA M1	37 (58.73)	21 (58.33)	16 (59.26)	
MCA M2	12 (19.05)	9 (25.00)	3 (11.11)	
Poor collateral circulation^$^	45 (71.43)	22 (61.11)	23 (85.19)	0.0363
Infarct core^*^, *ml*	12.0 (0.0, 50.0)	0.0 (0.0, 11.5)	55.0 (35.0, 98.0)	< 0.0001
CBV < 38%^*^, *ml*	16.0 (0.0, 57.0)	0.0 (0.0, 15.5)	71.0 (26.0, 104.0)	< 0.0001
Mismatch volume^*^, *ml*	111.0 (79.0, 160.0)	105.0 (80.0, 148.5)	128.0 (76.0, 238.0)	0.4285
Hypoperfusion^*^, *ml*	149.0 (100.0, 214.0)	116.0 (92.0, 151.0)	213.0 (151.0, 287.0)	0.0001
Tmax > 10 s^*^, *ml*	58.0 (21.0, 120.0)	40.50 (13.0, 62.50)	120.0 (73.0, 175.0)	< 0.0001
Mismatch ratio^*^	10.21 (3.02, 1000.00)	518.25 (9.29, 1000.00)	3.02 (1.91, 10.06)	< 0.0001
Hypoperfusion index^*^	0.45 (0.22, 0.58)	0.29 (0.12, 0.47)	0.57 (0.48, 0.64)	< 0.0001
Treatment^$^				
Antiplatelet therapy^$^	9 (14.29)	4 (11.11)	5 (18.52)	0.6606
Mechanical thrombectomy^$^	50 (79.37)	30 (83.33)	20 (74.07)	
Intravenous thrombolysis^$^	4 (6.35)	2 (5.56)	2 (7.41)	

^*^: Median (interquartile range)

^$^: *n*(%)

### Comparison of the three scoring methods

The comparison of the three scoring methods in patients with good and poor clinical outcomes is presented in Table 2. Significant differences were observed in the Auto-, NCCT- and CTP-ASPECTS between the patients with good and poor clinical outcomes (all *p* < 0.01). The NCCT-ASPECTS was higher than the other two scores. All the scores evaluated using the three methods in patients with poor clinical outcome were lower than those evaluated in patients with good clinical outcome. The proportion of patients with ASPECTS 0–5 in the poor clinical outcome group was significantly higher than that in the good clinical outcome group (all *p* < 0.01). Representative images from AIS patients with poor and good clinical outcomes are shown in Figs. 1 and 2, respectively.

**Table 2:** Comparison of the three scoring methods in patients with good and poor clinical outcomes

	Overall (*n* = 63)	Good clinical outcome (*n* = 36)	Poor clinical outcome (*n* = 27)	*p* value
*Ordinal scoring, M(IQR)*				
Auto-ASPECTS	8 (6, 9)	9 (7, 10)	7 (3, 9)	0.0070
NCCT-ASPECTS	10 (8, 10)	10 (9, 10)	8 (4, 10)	0.0006
CTP-ASPECTS	6 (4, 8)	8 (6, 9)	4 (1, 6)	< 0.0001
*Dichotomized scoring, n(%)*				
Auto-ASPECTS				
< 6	13 (20.63)	2 (5.56)	11 (40.74)	0.0006
≥ 6	50 (79.37)	34 (94.44)	16 (59.26)	
NCCT-ASPECTS				
< 6	9 (14.29)	0 (0.00)	9 (33.33)	0.0007
≥ 6	54 (85.71)	36 (100.00)	18 (66.67)	
CTP-ASPECTS				
< 6	23 (36.51)	3 (8.33)	20 (74.07)	< 0.0001
≥ 6	40 (63.49)	33 (91.67)	7 (25.93)	

**Fig. 3:**
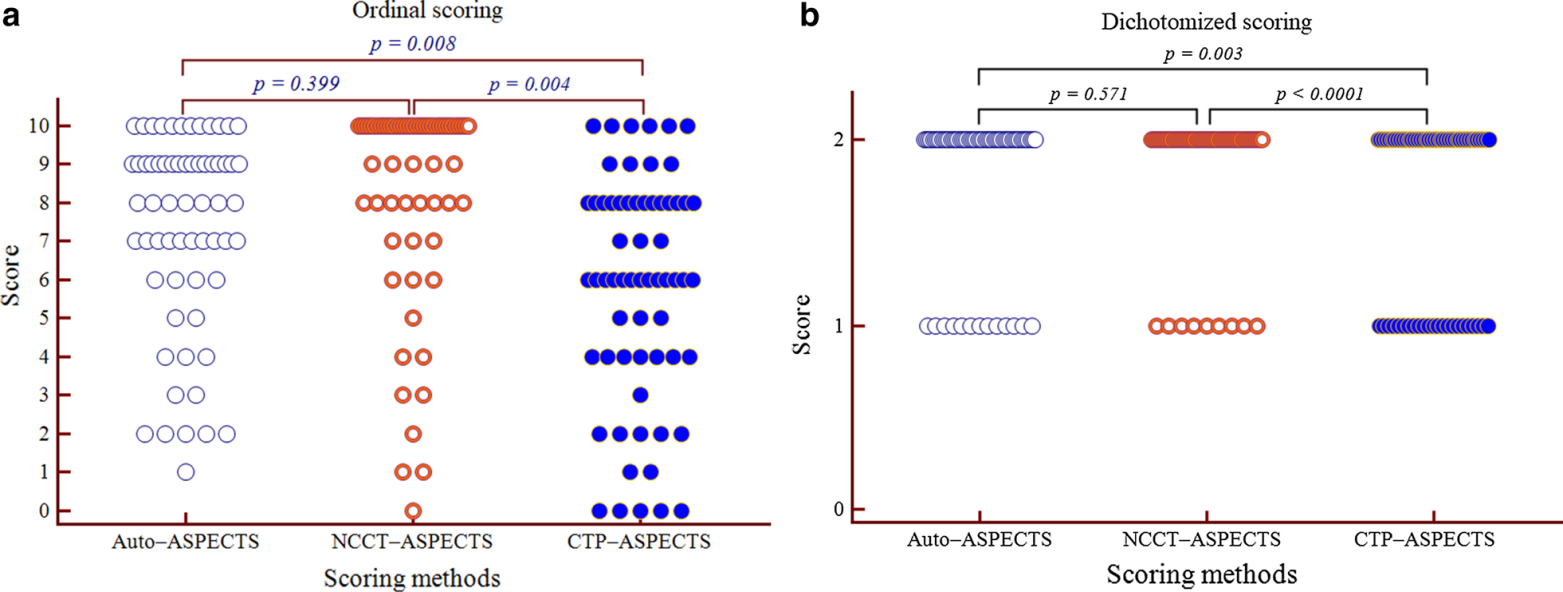
Comparative illustration of the distribution of the ordinal and dichotomized Auto-, NCCT- and CTP-ASPECTS in patients with AIS. Significant differences were observed in the ordinal (**a**) and dichotomized (**b**) scores among the three methods, while comparable scores were obtained using the Auto- and NCCT-ASPECTS methods

In addition, there were significant differences in the ordinal ASPECTS among the three methods and for the multiple comparisons (all *p* < 0.01) except for Auto- versus NCCT-ASPECTS (Fig. 3a). Significant differences were observed in the dichotomized ASPECTS among the three methods and for the multiple comparisons (all *p* < 0.01) except for Auto- versus NCCT-ASPECTS (Fig. 3b).

### Diagnostic performance of different scoring methods and clinical outcome

**Fig. 4:**
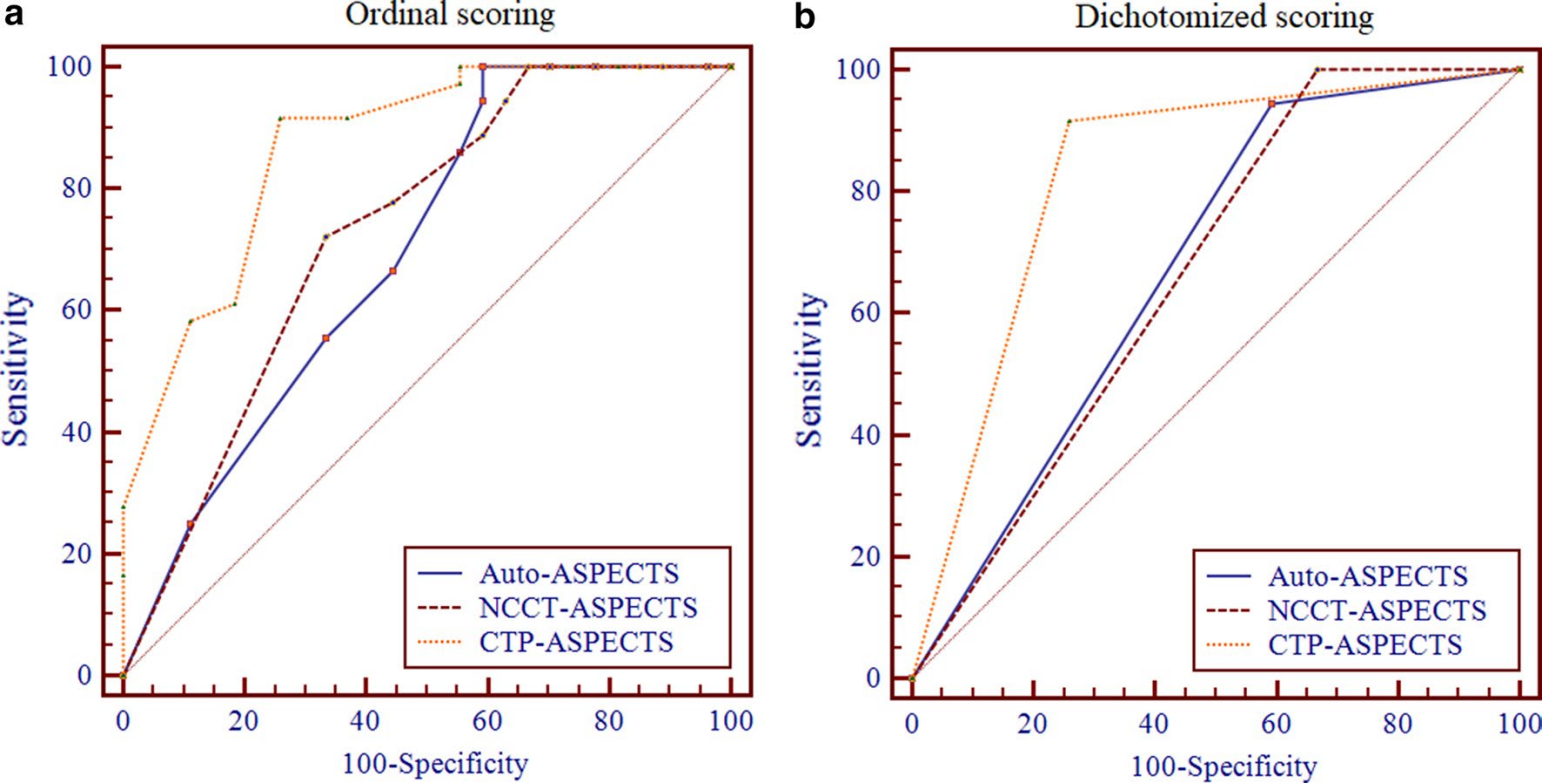
Receiver operating characteristic (ROC) curves for the ordinal and dichotomized Auto-, NCCT- and CTP-ASPECTS in patients with AIS. The areas under the curve (AUCs) of the ordinal Auto-, NCCT- and CTP-ASPECTS for predicting patients with good clinical outcome were 0.697, 0.730 and 0.870 (**a**), respectively. The AUCs of the dichotomized ASPECTS with a cutoff of > 5 was 0.676, 0.667 and 0.829 for the three scoring methods (**b**), respectively. Significant differences were observed between the areas of the ordinal or dichotomized CTP- and NCCT-ASPECTS and CTP- and Auto-ASPECTS, but no significant differences were observed between the areas of the ordinal or dichotomized Auto- and NCCT-ASPECTS

The diagnostic performance of the different scoring methods in patients with good and poor clinical outcomes is shown in Table 3. Figure 4 shows the ROC curves of the Auto-, NCCT- and CTP-ASPECTS for predicting the clinical outcome in patients with AIS. The areas under the curves (AUCs) of the ordinal Auto-, NCCT- and CTP-ASPECTS in predicting patients with good clinical outcome were 0.6970, 0.730 and 0.870, respectively. The AUCs of the dichotomized ASPECTS was 0.676, 0.667 and 0.829 for the three scoring methods, respectively.

**Table 3:** Diagnosis performance of different scoring methods in patients with good and poor clinical outcomes

	Auto-ASPECTS	NCCT-ASPECTS	CTP-ASPECTS
*Ordinal scoring*			
AUC	0.697	0.730	0.870
Cutoff value	> 4	> 9	> 5
Sensitivity	100.0	72.2	91.7
Specificity	40.7	66.7	74.1
PPV	69.2	74.3	82.5
NPV	100.0	64.3	87.0
*Dichotomized scoring*			
AUC	0.676	0.667	0.829
Cutoff value	> 5	> 5	> 5
Sensitivity	94.4	100.0	91.7
Specificity	40.7	33.3	74.1
PPV	67.9	66.7	82.5
NPV	84.6	100.0	87.5

*AUC* area under the curve, *NPV* negative predictive value, *PPV* positive predictive value

Moreover, the ROC curves for the ASPECTS from the three scoring methods were compared and summarized in Table 4. The AUCs of the ordinal CTP-ASPECTS was significantly higher than that of the NCCT- and Auto-ASPECTS (*p* = 0.0040 and 0.0008, respectively), with differences between the areas of 0.139 and 0.173, respectively. However, the difference between the areas of NCCT- and Auto-ASPECTS was 0.033, but no difference was found between the AUCs of them. Similar results were found using the dichotomized scoring methods. The AUCs of CTP-ASPECTS was higher than that of the other two methods (*p* = 0.0015 and 0.0025, respectively), with differences between the areas of 0.153 and 0.162, respectively, but the AUCs of the Auto-ASPECTS was similar to that of the NCCT-ASPECTS (*p* = 0.7730).

**Table 4:** Comparison of ROC curves in ASPECTS made by the three scoring methods

	Difference between area	SD	95% CI	*p* value
*Ordinal scoring*				
Auto- vs. NCCT-ASPECTS	0.033	0.040	-0.044–0.111	0.3990
Auto-vs. CTP-ASPECTS	0.173	0.052	0.072–0.274	0.0008
NCCT- vs. CTP-ASPECTS	0.139	0.048	0.045–0.234	0.0040
*Dichotomized scoring*				
Auto- vs. NCCT-ASPECTS	0.009	0.032	-0.054–0.072	0.7730
Auto- vs. CTP-ASPECTS	0.153	0.048	0.058–0.247	0.0015
NCCT- vs. CTP-ASPECTS	0.162	0.054	0.057–0.267	0.0025

*CI* confidence interval, *SD* standard deviation

## Discussion

This study assessed the diagnostic performance of the Auto-, NCCT- and CTP-ASPECTS for EICs in AIS patients with LVO, as well as explored which scoring method was most suitable for predicting the clinical outcome. For the ordinal and dichotomized scoring, significant differences were observed in the Auto-, NCCT- and CTP-ASPECTS between the patients with good and poor clinical outcomes. In addition, the CTP-ASPECTS was lower than the Auto- or NCCT-ASPECTS. Furthermore, the CTP-ASPECTS showed the best diagnostic performance among the three scoring methods in predicting the patients with good and poor clinical outcomes.

Several factors can affect the assessment of the ASPECTS, such as reader’s experience, training and the interval between symptom onset and CT examination [7, 11]. To improve the reader’s diagnostic ability, a narrow window width was used to enhance image contrast and improve sensitivity [3]. One study showed that the difference in the ASPECTS between the baseline NCCT using the optimized narrow window width and the follow-up magnetic resonance imaging (MRI) after treatment was small [16]. A prospective study of 214 patients with AIS or transient ischemic attack who underwent NCCT scans within 12 h of symptom onset showed that the NCCT-ASPECTS had good consistency between the physicians when performing real-time evaluations during CT scans [17]. It should be noted that the presence of terminus occlusion in the internal carotid artery and collateral strength must be considered when evaluating the ASPECTS, due to the acute occlusion in the distal IICA or proximal MCA with poor collateral strength may result in a low score [18].

CTP provides a more sensitive evaluation of EICs in AIS patients owing to it contains both physiologic and hemodynamic information about the ischemic tissue [19]. It offers several quantitative hemodynamic parameters, such as rCBV, rCBF and Tmax, which can reflect the perfusion status of ischemic brain tissue in AIS patients. It can identify potential low-risk perfusion territories, while EICs cannot be clearly shown on the early time window of the NCCT. Previous studies have shown that the CTP-ASPECTS is expected to improve the reliability of EICs evaluation and reduce the variability between observers compared with the NCCT-ASPECTS in the early time window [13, 20, 21].

This study showed that there was no significant difference between the NCCT- and Auto-ASPECTS, which is consistent with previous studies [7–9, 19, 22–24]. Sundaram et al. showed that the Auto-ASPECTS performed equally and the NCCT-ASPECTS in both the early and late time windows (≥ 6 or < 6 h) [19]. This shows that the Auto-ASPECTS has great potential in accelerating the evaluation of AIS patients, which could save time and reduce the burden of the doctors and may be widely applied in stroke centers at all levels.

In this study, the scores of the CTP-ASPECTS method were significantly lower than those of the other two scoring methods. The median CTP-ASPECTS was 6, which was lower than the previously reported 8, which might be due to the relatively high proportion of ASPECTS < 6 in the present study (23/63 vs 6/58) [19]. Since CTP can provide quantitative parameters such as the volume of the infarct core and ischemic penumbra, the CTP-ASPECTS may better reflect the true status of ischemic territory than the NCCT-ASPECTS.

As there is only low to moderate consistency between observers in the ASPECTS evaluation, neurologists often use the dichotomized ASPECTS for evaluating AIS patients, which may improve the consistency to moderate or good [11, 12, 25]. Desai et al. found that the ASPECTS ≥ 6 could be used to assess AIS patients in both early and late time windows (0–6 and 6–24 h) [26]. In this study, CTP-ASPECTS 0–5 accounted for 36.5% of the patients, which was higher than the other two methods, indicating that the CTP-ASPECTS might contribute to the highest identification of ischemic territory using the dichotomized ASPECTS.

The diagnostic performance of the CTP-ASPECTS was superior to that of the other two methods in predicting AIS patients with good clinical outcome. The AUCs of the ordinal CTP-ASPECTS was significantly higher than that of the Auto- and NCCT-ASPECTS, suggesting that the CTP-ASPECTS might be a better predictor of the clinical outcome than the other two scoring methods, with good sensitivity and moderate specificity. However, Sundaram et al. evaluated the performance of the Auto- against the NCCT-ASPECTS in a comparative analysis with concurrent CTP-ASPECTS and found no significant differences among the dichotomized NCCT-, Auto- and CTP-ASPECTS between patients with good and poor clinical outcomes, which was inconsistent with our results. The reason may be the low proportion of poor clinical outcome patients with ASPECTS 0–5 in their study [19]. Moreover, AIS patients with low ASPECTS may be associated with the poor clinical outcome [3, 27]. To select more AIS patients who will be more likely to benefit from different treatment strategies, three clinical trials excluded patients with low ASPECTS. The ESCAPE and SWIFT-PRIME trials excluded patients with a score < 6, while the REVASCAT trial excluded those with a score < 7 [28–30]. ASPECTS 6–10 was recommended for selecting AIS patients for mechanical thrombectomy [31]. In our study, ordinal or dichotomized CTP-ASPECTS 6–10 was used and showed good sensitivity (91.67%) and moderate specificity (74.07%) in predicting patients with good clinical outcome at 90 days follow-up. The specificity was higher than that of the Auto- and NCCT-ASPECTS.

There were several limitations in this study. First, it was a retrospective and single-center study, and further studies with larger sample sizes and more centers are needed to confirm the current findings. Second, only AIS patients with MCA and IICA occlusion were enrolled, which may result in the selection bias. Third, the final infarct volume could not be obtained to verify the accuracy of the Auto- and NCCT-ASPECTS because some patients hadn’t undergone the follow-up CT or MRI scans 24 h after the treatment; however, the CTP-ASPECTS might truly reflect the ischemic status at the scan session. Lastly, the heterogeneity induced by including patients treated with different strategies, however, there were no significant differences in treatment strategies (antiplatelet therapy, intravenous thrombolysis, and mechanical thrombectomy) between the patients with good and poor outcomes, we will enlarge the sample size and divide them into three subgroups: no reperfusion, thrombolysis only, and thrombectomy in the future study.

## Conclusion

The CTP-ASPECTS is superior to the Auto- and NCCT-ASPECTS in detecting EICs in AIS patients with LVO. The ordinal or dichotomized CTP-ASPECTS with a cutoff of 6 points is a good predictor of the clinical outcome at the 90-day follow-up.

## Data Availability

The datasets used and/or analyzed during the current study are available from the corresponding author on reasonable request.

## Abbreviations

AIS: Acute ischemic stroke
ASPECTS: Alberta stroke program early computed tomography score
AUC: Area under the curve
CTP: Computed tomography perfusion
EIC: Early ischemic change
MCA: Middle cerebral artery
mRS: Modified Rankin scale
NCCT: Noncontrast computed tomography
NIHSS: National institute of health stroke scale
